# Advances in 3D Printing: Microfabrication Techniques and Forming Applications

**DOI:** 10.3390/mi16080940

**Published:** 2025-08-15

**Authors:** Di Pan, Fanghui Jia, Muyuan Zhou, Hao Liu, Jingru Yan, Lisong Zhu, Ming Yang, Zhengyi Jiang

**Affiliations:** 1 School of Mechanical, Materials, Mechatronic and Biomedical Engineering, University of Wollongong, Wollongong, NSW 2522, Australia; 2 Graduate School of System Design, Tokyo Metropolitan University, 6-6-Asahigaoka, Hino 191-0055, Japan

**Keywords:** additive manufacturing, stainless-steel microfabrication, formability enhancement, post-processing

## Abstract

Stainless steel is essential in high-performance industries due to its strength, corrosion resistance, and biocompatibility. However, conventional manufacturing methods limit material efficiency, design complexity, and customization. Additive manufacturing (AM) has emerged as a powerful alternative, enabling the production of stainless-steel components with complex geometries, tailored microstructures, and integrated functionalities. Key AM methodologies, including laser powder bed fusion (L-PBF), binder jetting, and directed energy deposition (DED), are evaluated for their effectiveness in producing stainless-steel components with optimal performance characteristics. This review highlights innovations in stainless-steel AM, focusing on microfabrication, multi-material approaches, and post-processing strategies such as heat treatment, hot isostatic pressing (HIP), and surface finishing. It also examines the impact of process parameters on microstructure, mechanical anisotropy, and defects. Emerging trends include AM-specific alloy design, functionally graded structures, and AI-based control. Applications span biomedical implants, micro-tooling, energy systems, and automotive parts, with emphasis on microfabrication for biomedical micromachines and precision microforming.

## 1. Introduction

Stainless-steel remains the preferred material across high-performance sectors due to the mechanical strength, corrosion resistance, and biocompatibility. Traditional forming methods such as the subtractive machining, forging, and deep drawing are constrained by limited material efficiency, geometric complexity, and cost-effectiveness. As industries including biomedical, aerospace, and energy sectors increasingly demand miniaturised components with tailored functionality and complex geometries.

Additive manufacturing (AM) technologies represent a paradigm shift for stainless-steel fabrication. Laser powder bed fusion (L-PBF), binder jetting, and directed energy deposition (DED) have demonstrated capability for producing stainless-steel components with advanced geometric complexity, dimensional precision, and controlled microstructural characteristics. These layer-by-layer fabrication methodologies, driven from digital designs, substantially reduce material waste while eliminating traditional tooling constraints. Optimising grain orientation, phase distribution, and porosity allows tailoring of mechanical properties. Complementary post-processing protocols, particularly heat treatment and hot isostatic pressing (HIP), further enhance formability and surface integrity of stainless-steel components. These technological advances have facilitated deployment in critical applications, including patient-specific medical implants, conformal tooling solutions, and corrosion-resistant structures. Despite these advances, key challenges remain unresolved, including anisotropic mechanical behavior, residual stress accumulation, surface roughness, and reduced ductility.

This review examines recent innovations in stainless-steel AM, with emphasis on microfabrication strategies, post-processing methodologies, and their implications. The analysis identifies critical knowledge gaps and establishes research priorities for the integration of additively manufactured stainless-steel into broad forming technologies for possible applications on biomedical micromachines.

## 2. Microfabrication Approaches for Stainless-Steel

The successful fabrication of stainless-steel components via AM relies on the choice of processing technique and control of key parameters. Several microfabrication strategies have emerged as leading candidates for stainless-steel systems, each with distinct advantages in terms of resolution, productivity, and suitability for forming-related applications.

### 2.1. L-PBF

L-PBF has emerged as a cornerstone technology for stainless-steel AM, delivering components with exceptional geometric, near-full density, and superior surface finish.

**Figure 1:**
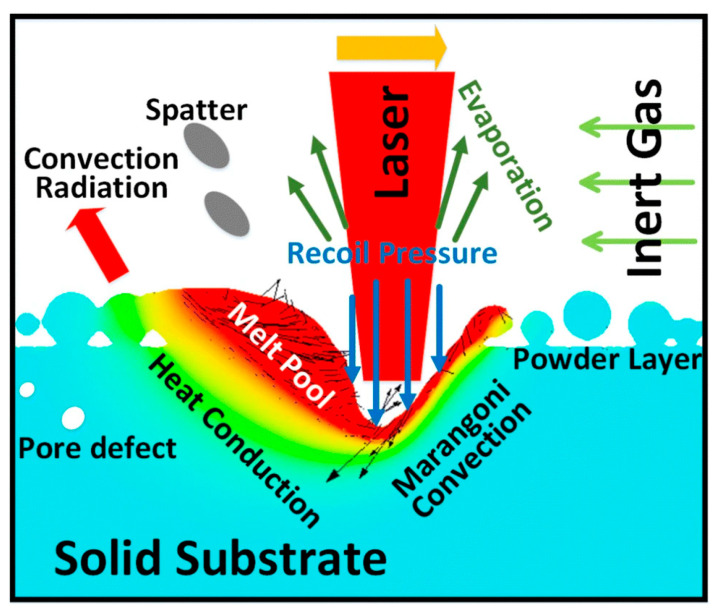
Schematic plot of the general phenomena in the L-PBF [1].

**Figure 2:**
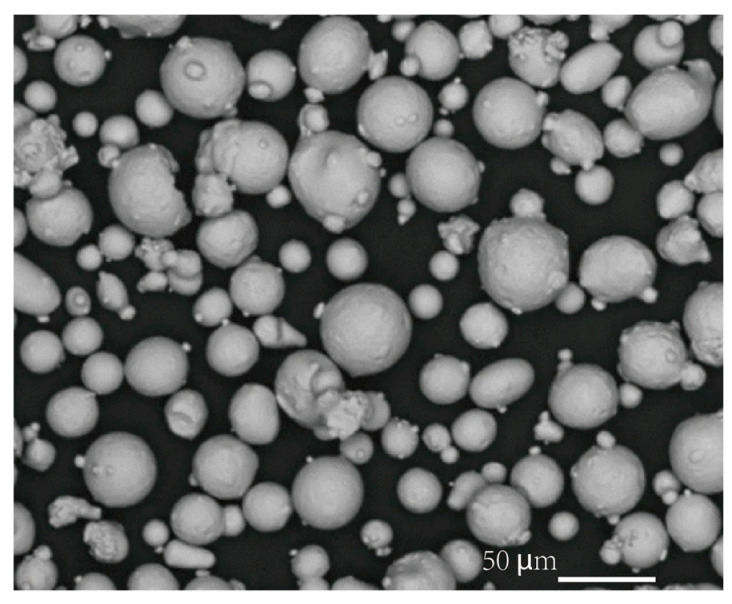
Metallic powder of stainless steel 316L [2].

Figure 1 presents a schematic illustration of the general phenomena in L-PBF. During fabrication, metal powder is spread uniformly across the build platform, and a focused laser selectively melts regions per the digital model. After solidification, the platform lowers and the process repeats, constructing a fully dense three-dimensional object. Precise powder characteristics—morphology, particle size distribution, and flowability—are critical for repeatable layer quality and defect-free builds. Figure 2 illustrates the stainless steel 316L powder used in L-PBF processing. The laser navigates across the powder bed following digital cross-sectional data, repeating layer upon layer until completing the final component.

**Figure 3:**
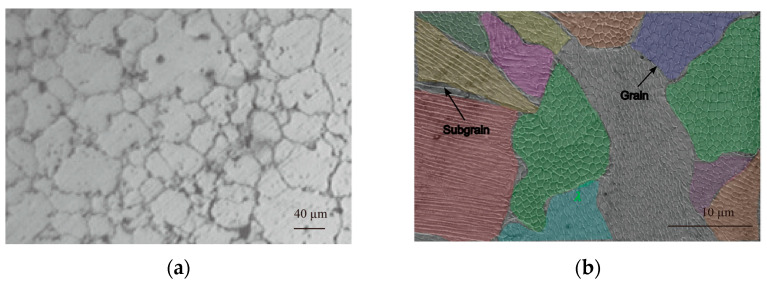
(**a**) Optical micrographs of stainless steel 316L [4]; (**b**) additively manufactured samples for microstructure [2].

Austenitic stainless-steels such as 316L and precipitation-hardened grades like 17-4PH achieve densities exceeding 99.5% through L-PBF, exhibiting submicron grain sizes and minimal residual porosity when processing parameters are optimised [2,3]. Figure 3 shows (a) optical micrographs of stainless steel 316L and (b) additively manufactured samples for microstructure [2]. The rapid solidification rates generate refined cellular grain structures, enhancing yield strength and hardness compared with conventionally manufactured counterparts. This distinctive microstructure, combined with exceptional dimensional precision, establishes L-PBF as a superior manufacturing process.

**Figure 4:**
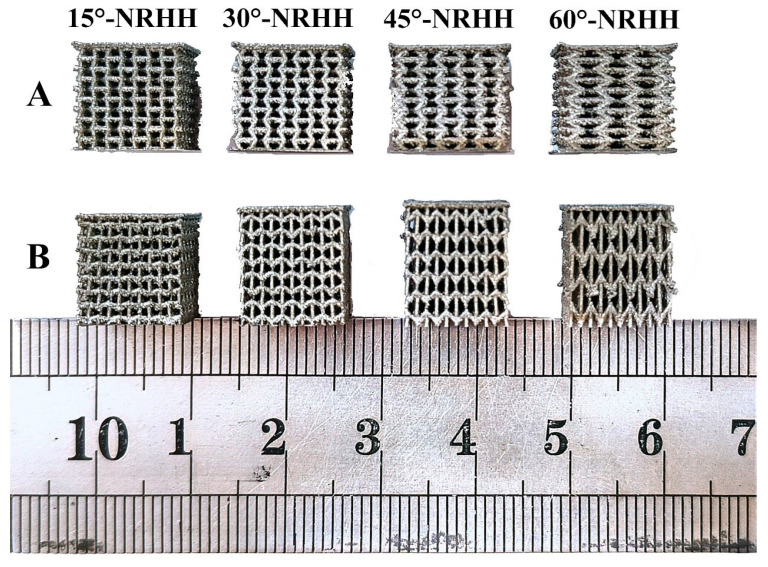
SLM prepared porous skeletal scaffolds with different structures of NRHH, group A was molded with Y-axis as the construction direction and group B was molded with Z-axis as the construction direction [5].

These application-specific examples highlight the material-process synergy of L-PBF with 316L stainless steel, demonstrating its viability for industrial, biomedical, and tooling sectors requiring high geometric complexity, material performance, and customisation. Figure 4 shows L-PBF-fabricated 316L porous scaffolds with 75% porosity and 400–700 μm pores, ideal for bone ingrowth and vascularisation. Porous orthopedic scaffolds are designed with controlled pore sizes (300–600 µm) and interconnected porosity to mimic cancellous bone structure. These implants facilitate tissue ingrowth and vascularisation, while L-PBF enables precise control over porosity gradients and lattice topology, critical for mechanical compatibility and biological performance. Two build orientations—Y-axis (Group A) and Z-axis (Group B)—were used to tailor the negative Poisson’s ratio (NPR) behaviour. Y-oriented scaffolds enhanced lateral compliance, while Z-oriented designs improved axial flexibility, enabling direction-specific mechanical tuning. This is valuable for orthopedic implants requiring site-specific deformation, such as spinal cages or femoral stems. Porous orthopedic micro scaffolds with controlled pore sizes (300–600 µm) and interconnected porosity facilitate tissue ingrowth and vascularisation, demonstrating the micro-scale precision achievable with L-PBF.

**Figure 5:**
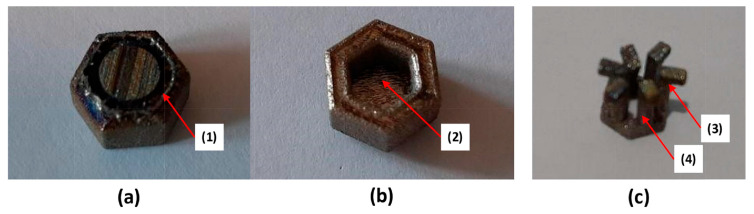
L-PBF fabricated snap-fit abutment elements: (**a**) upper section—top view, (**b**) upper section—bottom view, (**c**) lower section [6].

**Figure 6:**
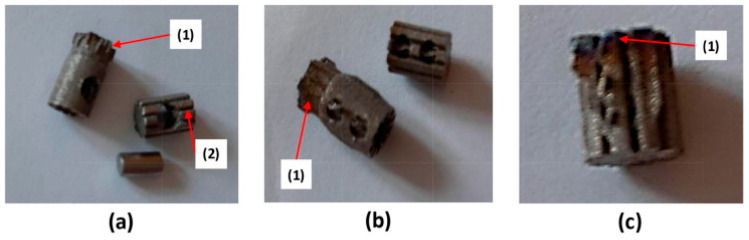
Spline connection abutments manufactured via L-PBF: (**a**) single-pin design, (**b**) dual-pin design, (**c**) triple-pin design [6].

The dental abutments were produced using an SLM280HL system (SLM Solutions AG, Germany) operating within a pure argon atmosphere containing less than 0.1% oxygen. The feedstock material consisted of Grade 2 commercially pure titanium powder (TLS Technik, Germany) characterised by spherical particle morphology and a size distribution ranging from 20 to 65 μm. Manufacturing was performed using the following operational parameters: laser power of 275 W, scan velocity of 1100 mm/s, hatch distance of 0.120 mm, and powder layer thickness of 30 μm. The manufacturing challenges and defects encountered during abutment production are illustrated in Figure 5 and Figure 6. For the snap-fit design, challenges include: (1) support structures trapped between features that cannot be removed, (2) overhanging sections that require supports during fabrication, leading to dimensional deviations and reduced manufacturability, (3) cantilever beams that are stiffer than intended due to variations in L-PBF material properties, and (4) bending difficulties caused by the overall size and feature dimensions of the abutment. In the spline connection designs, adequate clearance between the top and bottom parts is necessary to ensure proper alignment and fitting. Additional common issues involve (1) grooves too small to be accurately produced by L-PBF and (2) geometric inaccuracies arising from fine feature sizes. For instance, in the three-pin spline connection design, the pin holes are smaller than intended, making the removal of support structures difficult. Micro-sized dental abutments produced via L-PBF illustrate the importance of achieving precise micro-features critical for dental micromachine applications.

The quality of L-PBF fabricated components demonstrates high sensitivity to numerous process parameters. Laser power, scan speed, hatch spacing, layer thickness, scan strategy, and powder characteristics influence thermal gradients, cooling rates, and melt pool morphology, ultimately determining microstructural evolution and defect formation [7,8]. Low laser energy densities result in incomplete fusion, creating lack-of-fusion pores that compromise mechanical strength. High energy densities may generate keyhole porosity, where vaporised material traps gas bubbles. Improper scan overlap can create inconsistent bonding, forming weak interlayer adhesion and localised stress concentrations [9]. L-PBF intrinsically induces directional solidification patterns, with grains growing preferentially along the build direction (Z-axis). This phenomenon produces mechanical anisotropy, where the tensile strength and elongation differ between horizontal and vertical orientations. Forming applications utilising such materials may experience non-uniform deformation, premature failure, or accelerated tool wear without appropriate design considerations [10]. Recent advances in in-situ monitoring systems have enhanced the reliability of L-PBF for stainless-steel fabrication. Techniques such as melt pool imaging, acoustic feedback, and adaptive parameter control allow for real-time quality assurance and process optimisation. These developments have reinforced the suitability of L-PBF for producing components used in precision forming, micro deep drawing, and advanced tooling systems [11]. These technological developments significantly reduce process variability, enhance reproducibility, and enable tailored performance characteristics meeting application-specific requirements.

### 2.2. Binder Jetting

Binder jetting has emerged as a promising AM technique for producing stainless-steel components with high throughput, low cost, and scalable production potential.

**Figure 7:**
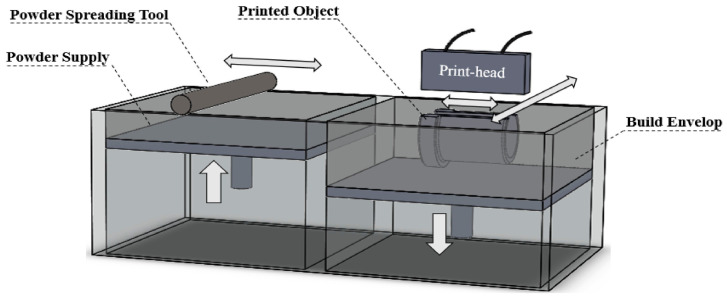
Binder jetting system schematic [20].

Figure 7 shows a standard binder jetting where powder is spread by a roller, then a print head selectively deposits liquid binder to form each layer’s pattern. The build platform lowers after each layer, and the process repeats. Optional heating may control moisture and curing. The printed parts are initially fragile and require post-processing to improve mechanical properties. In contrast to fusion-based methods such as L-PBF, binder jetting follows a two-step process. First, a liquid binder is selectively deposited onto a bed of stainless-steel powder to form a loosely bound “green part.” This is followed by thermal debinding and high-temperature sintering to achieve final densification and metallurgical bonding [12,13]. This method offers several advantages over laser-based systems. It enables fast build rates, large component volumes, and low residual stress due to the absence of localised melting. Consequently, binder jetting is well-suited for producing near-net-shape components, customised forming dies, and microscale bulk structures where dimensional tolerance is critical, but ultra-high strength is not the primary requirement [14,15]. Additionally, its compatibility with multi-part nesting and powder recyclability enhances its economic feasibility for batch manufacturing of stainless-steel components. However, binder jetting presents its own set of technical challenges that must be carefully addressed. The initial green part typically exhibits low strength and high porosity, making it susceptible to distortion during subsequent sintering processes. The sintering step is critical in defining the microstructure, density, and mechanical performance of the component. Parameters such as temperature, dwell time, and furnace atmosphere influence pore elimination, grain growth, grain boundary evolution, and shrinkage uniformity [16,17]. For stainless-steels like 316L, binder jetting achieves 95–98% relative density after sintering [18]. While this density is lower than that in the L-PBF processes, the mechanical properties are often sufficient for tooling and structural applications. However, parts may exhibit coarser grains, lower tensile strength, and reduced ductility, along with higher surface roughness. To overcome these inherent limitations, researchers have developed comprehensive optimisation strategies. Liquid-phase sintering additives such as boron and phosphorus are employed to promote densification and enhance grain boundary wetting, creating transient liquid phases that facilitate particle rearrangement and reduce required sintering temperatures [19]. Table 1 show that higher sintering temperatures (1300 to 1380 °C) improved mechanical properties by reducing porosity. Tensile strength increased from 323.4 ± 22.9 MPa to 473.7 ± 7.3 MPa, while elongation rose from 6.1% to 40.2%.

**Table 1:** Mechanical properties of sintered 316L with different sintering temperatures [15].

	1300 °C	1340 °C	1360 °C	1380 °C	1400 °C
Ultimate tensile strength (MPa)	323.4 ± 22.9	368.7 ± 28.4	423.3 ± 27.6	473.3 ± 7.3	387.0 ± 21.8
Yield strength (MPa)	185.9 ± 3.2	171.8 ± 3.9	186.4 ± 4.9	182.7 ± 3.8	247.6 ± 17.1
Elongation at break (%)	6.1 ± 3.2	16.8 ± 2.2	26.2 ± 3.3	40.2 ± 2.4	10.6 ± 2.8

**Figure 8:**
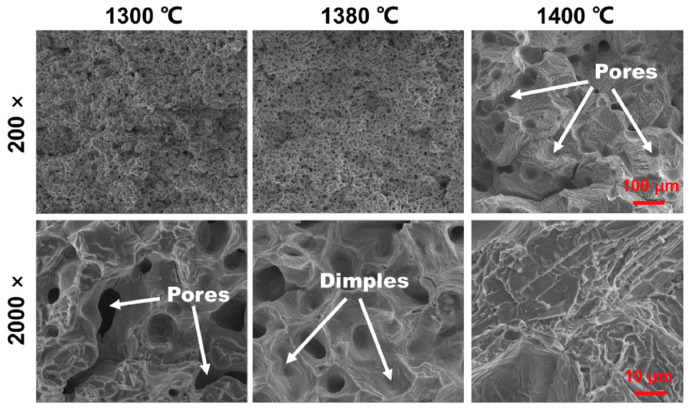
Fracture surface morphologies of tensile-tested 316 L specimens sintered at 1300 and 1380 °C [15].

**Figure 9:**
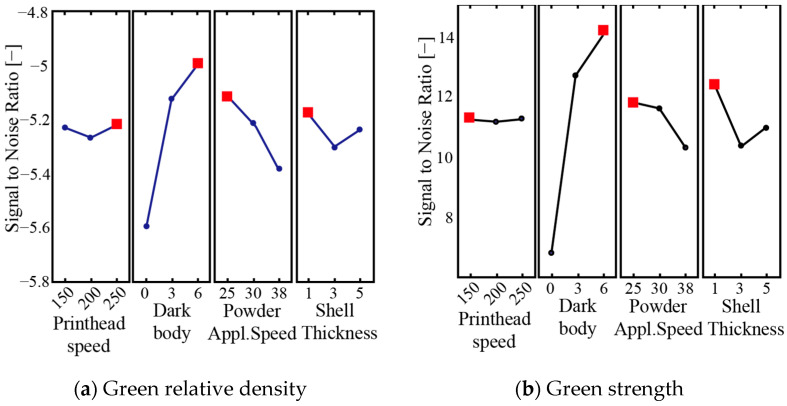
Signal-to-noise ratios for (**a**) relative density and (**b**) green state bending strength [18].

Porosity negatively affects strength through two mechanisms: reducing the load-bearing area and creating stress concentrations that cause premature failure. Figure 8 displays the fracture surface characteristics of tensile specimens sintered at 1300 and 1380 °C. Lower porosity at higher temperatures reduces crack initiation sites, enabling greater deformation. Fracture analysis confirmed that samples sintered at 1380 °C showed deep dimples and few pores, indicating more plastic deformation. However, samples sintered at 1400 °C showed decreased properties (tensile strength dropped to 387.0 ± 21.8 MPa, elongation to 10.6%) due to excess carbon content from incomplete debinding and excessive grain growth. The fracture morphology revealed intergranular brittle failure with minimal dimples, confirming the loss of ductility at this temperature. Additionally, binder system tuning involves careful adjustment of solvent ratios and polymer compositions to enhance green part strength while minimising warping and dimensional distortion during the debinding process [15]. According to Figure 9, printhead speed showed the smallest effect and was used as an error term to calculate Fisher (F) and probability (*p*) values. Using a *p*-value threshold of 0.1 to determine statistical significance, the analysis revealed that dark body parameters significantly influence both relative density and green strength. Powder applicator speed also significantly affects relative density. These findings align with previously published research.

**Figure 10:**
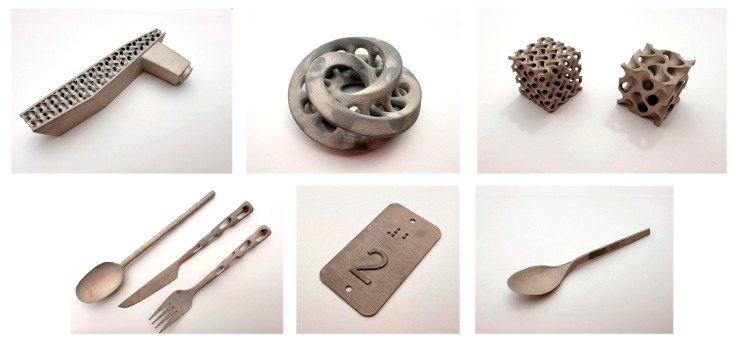
Representative components produced via binder jetting technology [21].

Figure 10 presents representative components fabricated using binder jetting technology. In forming applications, binder-jetted stainless-steel tools provide distinct advantages, especially for tasks that demand complex geometries, intricate internal channels, custom profiles, and precise dimensional accuracy. This technology is particularly suitable for short-run production or prototyping phases, where rapid turnaround and lower upfront costs significantly benefit manufacturing processes. Although the mechanical properties of binder-jetted components typically do not match those fabricated through L-PBF, the inherent flexibility and economic efficiency offer a highly beneficial trade-off. Consequently, binder jetting has become increasingly attractive for low-to-medium load tooling applications, such as adaptive forming inserts, precision molds, dies, and fixtures commonly employed in micro-manufacturing systems. Furthermore, with advancements in sintering optimization, post-processing strategies, and novel alloy formulations, binder jetting continues to improve, broadening its applicability and performance in manufacturing sectors demanding tailored functionality and cost-effective precision.

### 2.3. DED

**Figure 11:**
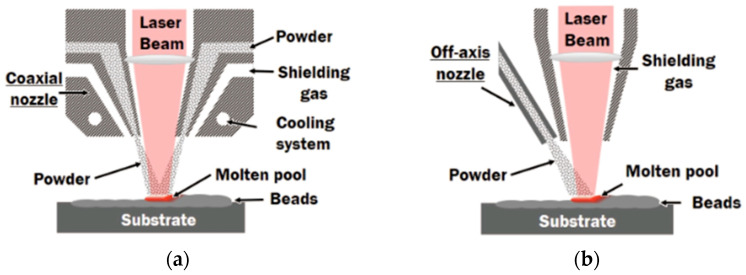
Powder delivery approaches in DED: (**a**) coaxial powder injection and (**b**) lateral powder injection [30].

**Figure 12:**
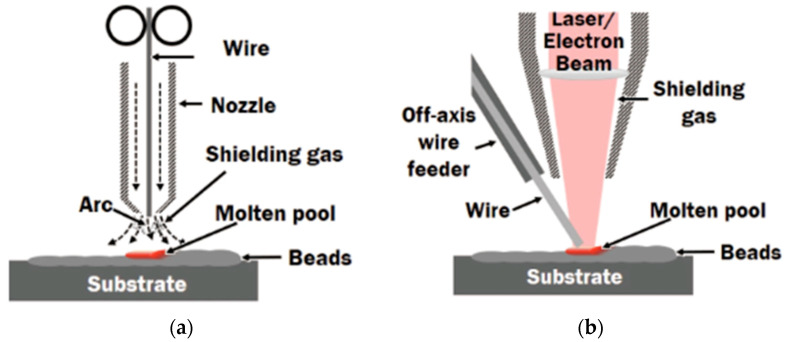
Wire delivery approaches in directed energy deposition systems: (**a**) coaxial wire feeding and (**b**) lateral wire feeding [30].

DED is a powerful and flexible AM process in which metal powder or wire is fed directly into a molten pool created by a concentrated energy source, typically a laser, electron beam, or plasma arc. The operational mechanism of the DED process is demonstrated in Figure 11 and Figure 12. This AM employs principles derived from cladding and welding methodologies. A concentrated thermal energy source—including laser, electron beam, or welding heat flux—is directed onto the previously deposited layer. Concurrently, feedstock material, available in wire or powder form, is introduced into the thermal energy focal zone. The convergence of thermal energy with both the substrate layer and incoming feedstock creates a molten pool within and surrounding the energy application point. As this molten pool solidifies, it forms a deposited track. Through systematic repetition of this deposition cycle, three-dimensional metallic components are constructed layer by layer. The process is characterised by rapid solidification, with typical cooling rates spanning 10^3^ to 10^5^ °C/s. Unlike powder bed-based methods such as L-PBF, DED operates with an open architecture and multi-axis deposition head, allowing it to fabricate large parts, repair damaged components, and deposit materials onto existing substrates with minimal constraints on geometry [22,23].

In DED, metal feedstock is delivered through coaxial and simultaneously melted by the energy beam. The process builds components layer-by-layer, like traditional AM systems, but with greater flexibility in Z-axis, material composition, and substrate compatibility. For stainless-steel systems such as 316L, 17-4PH, or duplex grades, DED offers unique capabilities that are beneficial for applications. Site-specific deposition enables selective reinforcement of high-wear zones such as punch edges, draw bead regions, or die corners. Functionally graded materials (FGMs) can be fabricated by gradually varying alloy composition, creating property gradients within a single component. Additionally, bimetallic deposition enhances thermal conductivity, surface hardness, and corrosion resistance, depending on forming requirements [24,25]. The process parameters in DED, including laser power, powder feed rate, traverse speed, layer height, and shielding gas flow, must be controlled to ensure stable melt pool dynamics, strong interlayer bonding, and uniform microstructure. Improper tuning may result in high dilution of the substrate, excessive heat input leading to grain coarsening, residual stresses, and weak interfaces between dissimilar materials [26,27]. In stainless-steel DED, microstructure control is often achieved through controlled cooling strategies or thermal feedback systems, which can promote fine grain structures and reduce anisotropy. Moreover, closed-loop control systems and multi-material deposition heads are pushing DED close to a fully automated, intelligent fabrication method for functional forming systems [28,29]. Previous experimental work established optimal laser powers of 800 W for pure 316L and 1800 W for pure Ti6Al4V fabrication.

**Figure 13:**
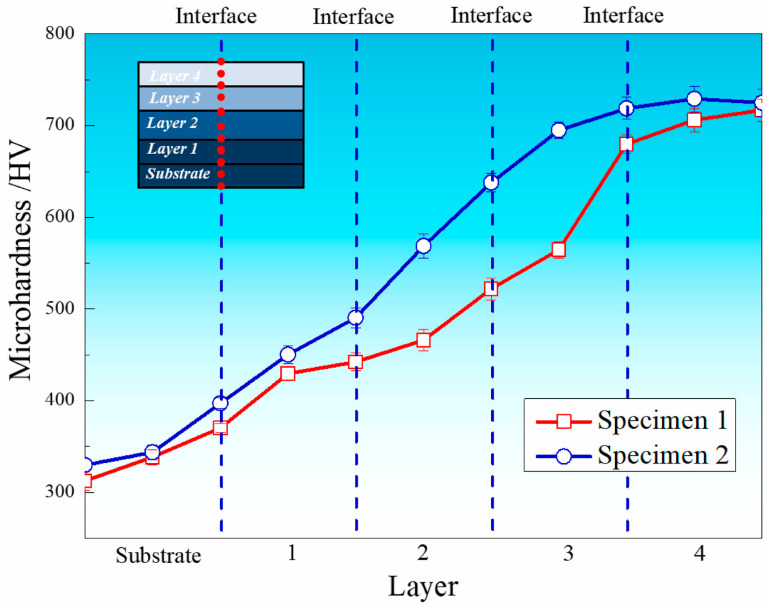
Microhardness of different layers in both specimens [32].

**Figure 14:**
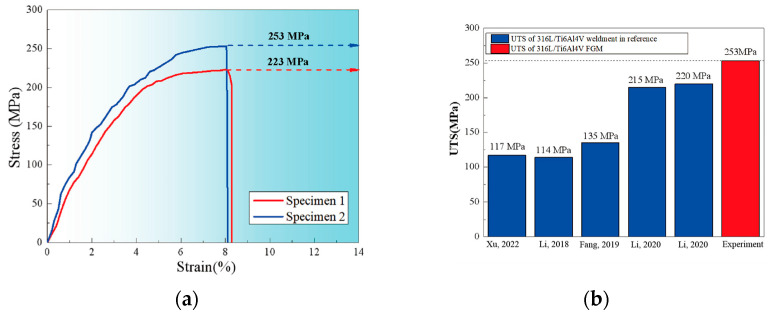
(**a**) Engineering stress-strain curves under tensile loading, and (**b**) Ultimate tensile strength comparison between 316L/Ti6Al4V FGM and 316L/Ti6Al4V bimaterials produced using various manufacturing techniques [32,33,34,35,36].

As the proportion of 316L increases in the material composition, lower laser power is required for effective powder melting. Li et al. [31] demonstrated that excessive laser power results in molten pool instability during DED. When a uniform 1800 W laser power was applied across all layers, the surplus energy input compromised molten pool stability, particularly in regions with higher 316L content. Xu et al. [32] investigated functionally graded Ti6Al4V/316L specimens fabricated using different laser power strategies. Specimen 1 was produced using a constant laser power of 1800 W, while Specimen 2 employed a decreasing laser power approach, reducing from 1800 to 1200 W across the Ti6Al4V to 316L gradient [32]. Figure 13 demonstrates the progressive increase in microhardness across the graded layers in both specimens. For Specimen 1, microhardness measurements reveal values of approximately 338 HV in the substrate, followed by 429 HV, 466 HV, 570 HV, and 706 HV in the four successive deposited layers. Specimen 2 exhibits enhanced microhardness performance, with values ranging from 343 HV in the substrate to 729 HV in the fourth layer. Notably, Specimen 2 consistently demonstrates superior microhardness compared to Specimen 1 at equivalent layer positions. Beyond the microhardness improvements, the overall tensile behaviour of Ti6Al4V/316L FGMs requires evaluation. Figure 14a presents the engineering stress-strain curves for both specimen types. Specimen 1 achieved an ultimate tensile strength (UTS) of 223 MPa with 8.3% strain, while Specimen 2 demonstrated superior performance with a UTS of 253 MPa and 8.1% strain. This represents a 13.5% improvement in tensile strength for Specimen 2 compared to Specimen 1, confirming that variable laser power strategies enhance the mechanical properties of FGMs. Nevertheless, as illustrated in Figure 14b, the strength of DED-fabricated 316L/Ti6Al4V FGMs produced using gradient laser power exceeds that of 316L/Ti6Al4V bimaterials manufactured through conventional welding processes [33,34,35,36]. DED is particularly advantageous for hybrid manufacturing, where it is combined with subtractive machining. This integration allows for geometric correction, precision feature integration, and surface finish enhancement [37]. Despite these advantages, DED offers lower resolution and surface quality compared to L-PBF. The resulting surface roughness, typically in the range of 10–25 µm, may require post-processing such as CNC machining, grinding, or shot peening for forming applications. However, its high deposition rates (10–100 cm^3^/h) and flexibility in geometry make it attractive for repair, rework, and tooling augmentation [38].

**Figure 15:**
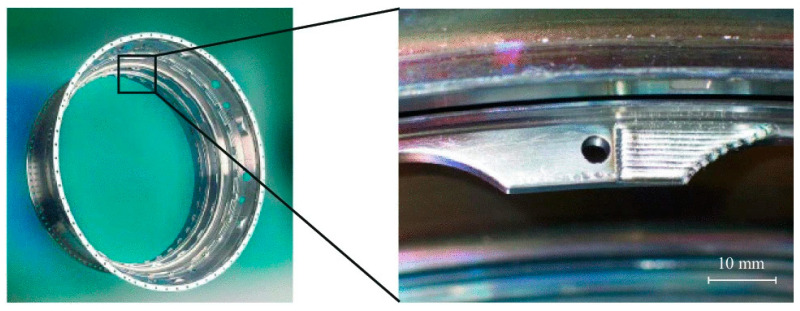
Restoration of a BR715 HPT case flange via laser metal deposition techniqu [39].

**Figure 16:**
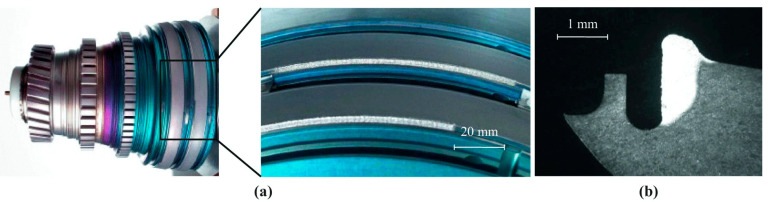
(**a**) Repair of a Ti6Al4V groove wall using the LP-DED process; (**b**) Cross-sectional view of the restored groove [39].

The DED process has received certification for 15 distinct repair procedures [39]. Two notable examples of its application are illustrated in Figure 15 and Figure 16. In Figure 15, a BR715 high-pressure turbine casing made from a nickel alloy is shown. Specific regions of this component—such as bosses, flanges, and brackets—experience wear during operation. DED was effectively used to restore a worn flange made from the nickel-based Nimonic PE16 alloy, using Ni625 powder as the repair material. The process was performed under shielding gas to prevent oxidation. Figure 16 depicts a repair operation on the damping wire grooves of a BR715 high-pressure compressor front dump constructed from Ti6Al4V alloy. This repair involved localised restoration of worn groove walls, which posed two main challenges: ensuring that the opposite wall remained unaffected and working within the spatial constraints imposed by the groove geometry. Despite these difficulties, LP-DED successfully addressed the issues, and further refinement of process parameters is needed to enhance repair quality [40]. In a related study, Li et al. [41] explored the use of LP-DED for repairing aluminum alloy aircraft structures. Their preliminary findings revealed that the process could achieve sound metallurgical bonds without cracking when optimised parameters were used. However, the interface between the deposited layer and the substrate remained a weak point, resulting in reduced tensile strength and fatigue life compared to the original material. Thus, continued research is necessary to fully optimise the repair performance.

Recent advancements in DED have also enabled the fabrication of FGMs, such as 316L/Inconel systems, which are of particular interest for aerospace and nuclear applications. These FGMs leverage the room-temperature ductility and low-temperature strength of stainless steel 316L, combined with the high-temperature strength and oxidation resistance of Inconel alloys. However, challenges arise due to the formation of brittle intermetallic phases at the interface, often resulting in cracking. To address this, advanced DED strategies such as local composition detouring have been proposed. This approach modifies the composition pathway to bypass deleterious phase regions in the binary phase diagram, effectively mitigating crack formation and enabling defect-free graded structures [42].

## 3. Forming Behaviour and Post-Processing

Post-processing plays a crucial role in tailoring the mechanical properties, microstructural stability, and surface quality of stainless-steel components fabricated through AM. While AM techniques such as L-PBF, DED, and binder jetting offer design and fabrication flexibility, the as-printed parts exhibit residual stresses, porosity, anisotropic mechanical properties, and surface defects.

### 3.1. Forming Performance of AM Stainless-Steel Components

While AM of stainless-steel enables advanced geometries and tailored microstructures, challenges persist in forming-intensive applications including microforming, bending, deep drawing, and incremental sheet forming. These limitations stem from intrinsic characteristics of AM-fabricated components, including anisotropic grain structures, reduced ductility, and surface-related imperfections. The elongation and fracture toughness reduction primarily results from microsegregation, non-equilibrium solidification, and fine subgrain structures that restrict dislocation mobility and facilitate early strain localisation [43]. Research examining L-PBF 316L and 17-4PH indicates elongation values 15–30% lower than conventionally rolled products, increasing failure risk during forming operations [44].

Layer-wise deposition and directional thermal gradients during AM processing create texture development and mechanical anisotropy, particularly between *Z*-axis and planar (XY) directions. The *Z*-axis frequently exhibits lower ductility and yield strength due to poor interlayer bonding and thermally induced defects [45]. During forming processes, this anisotropy produces non-uniform deformation zones, asymmetric springback, and localised thinning [46]. Even with extensive post-processing, complete microstructural homogenisation rarely occurs, rendering AM component forming behaviour orientation sensitive. The rough, porous, and particle-adhered surface of AM components functions as stress risers during plastic deformation. For microforming applications, where surface-to-volume ratios remain high, these defects cause early crack initiation, friction-induced surface damage, and wrinkling with non-uniform material flow [47]. Even following post-processing treatments such as electropolishing or AFM, subsurface defects persist and degrade component tribological and mechanical integrity. This anisotropy significantly influences microforming processes such as micro deep drawing and micro-stamping, where uniform deformation and micro-scale precision are critical.

Recent studies explore several approaches to improve forming performance. Warm forming at elevated temperatures (200–400 °C) reduces yield stress while increasing ductility for AM 316L and 17-4PH [48]. Multi-step forming with interpass annealing prevents strain localisation accumulation and enhances uniform deformation [49]. Ultrafine grain tuning via thermo-mechanical cycling refines grain size, enhances ductility, and homogenises strain distribution throughout components. Advances in predictive simulation, in-situ monitoring, and data-driven process control enable improved correlation between AM process conditions, microstructural features, and forming performance. As process standardisation and material databases mature, AM stainless-steels are expected to meet requirements for high-precision forming applications across automotive, aerospace, and biomedical tooling domains.

### 3.2. Heat Treatment and Microstructural Evolution

Heat treatment represents an essential post-processing strategy for additively manufactured stainless-steels, serving to optimise microstructure, phase composition, and mechanical performance for subsequent forming operations. The rapid solidification and thermal gradients inherent to AM processes produce stainless-steel components with non-equilibrium microstructures, directional grain growth, and elevated residual stress levels, limiting ductility and formability. Heat treatments assume a central role in enhancing performance through microstructural homogenisation and stress relaxation [50].

**Figure 17:**
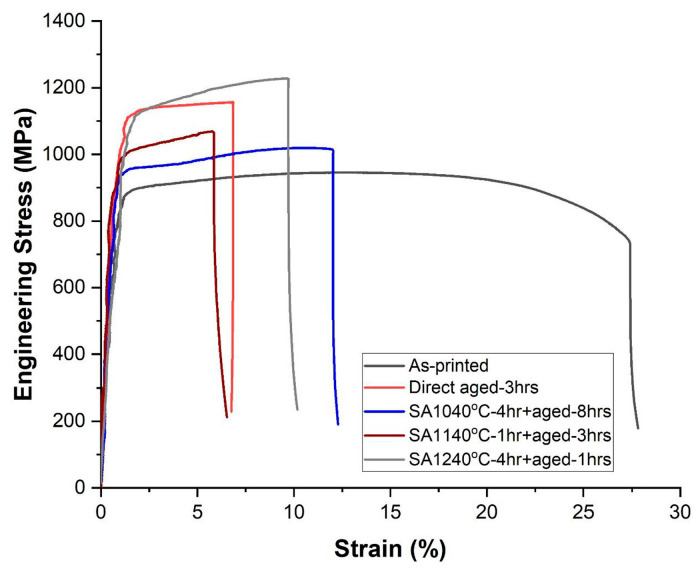
Tensile stress-strain curves comparing as-printed and heat-treated SLM 17-4PH steel [58].

Austenitic stainless-steels like 316L undergo solution annealing at temperatures ranging from 1050 to 1100 °C. This treatment promotes secondary phase dissolution, concurrently alleviating chemical segregation and facilitating the transformation from elongated columnar grains to equiaxed grain structures. Consequently, mechanical anisotropy decreases substantially, while elongation and formability improves significantly [51,52]. Precipitation-hardenable stainless-steels such as 17-4PH require multi-step thermal cycles for optimal performance. Solution treatment occurs at approximately 1038 °C to dissolve existing precipitates and homogenise the matrix. Subsequent aging treatments between 480 and 620 °C precipitate Cu-rich strengthening phases that increase hardness, yield strength, and creep resistance. The aging temperature and duration determine precipitate morphology, distribution patterns, and mechanical performance [53]. Both alloy systems frequently employ stress-relief annealing as preliminary treatment to reduce residual tensile stresses. Figure 17 shows that heat-treated samples had higher strength but lower ductility than as-printed samples. Direct aging at 480 °C increased ultimate tensile strength from 914 to 1184 MPa due to copper-rich precipitates and austenite-to-martensite transformation, but reduced elongation from 20.2 to 8.3%. The strongest condition (solution treatment + aging) achieved ~1200 MPa with only 4.4% austenite remaining. While as-printed samples showed significant necking, heat-treated samples fractured immediately at peak stress. The ~30% strength increase in solution-treated material resulted from reduced austenite, copper-rich precipitates, and carbide formation. The heat treatment proves especially critical for components undergoing cold forming, micro deep drawing, or fatigue loading, where residual stresses may initiate dimensional instability [54]. Thermal treatments influence grain boundary character distribution by promoting the formation of low-energy grain boundaries, which enhance the material’s resistance to intergranular cracking and environmentally assisted degradation mechanisms. These improvements are particularly crucial for applications such as forming dies or biomedical components that must withstand complex loading conditions and corrosive environments during service [55]. Advanced methodologies, including multi-step thermal cycling, double aging, and laser-assisted annealing, are currently under investigation to further optimise property gradients, improve thermal stability, and extend tool life under dynamic forming conditions. Additionally, combining heat treatment with thermo-mechanical processing or HIP represents a promising strategy to simultaneously densify, refine, and toughen AM-fabricated stainless-steel components [56,57].

HIP application increasingly occurs alongside heat treatment, producing synergistic improvements in microstructure, hardness, and isotropy. HIP serves as a powerful volumetric densification method for eliminating internal porosity in AM stainless-steels. This process exposes components to high pressures (100–200 MPa) and elevated temperatures (1150–1250 °C) within inert gas environments, typically argon. Under these conditions, diffusion mechanisms activate, enabling pore closure, void elimination, and enhanced interparticle bonding [59]. HIP proves valuable for binder jetting and L-PBF-produced stainless-steel due to high porosity levels (~2–5%) often retained after sintering. HIP-treated components exhibit increased bulk density, enhanced fatigue resistance and fracture toughness, and reduced crack initiation likelihood in high-stress zones of forming dies. These improvements contribute to higher formability indices, particularly important for deep drawing, stamping [60]. Two groups of 316L stainless steel samples were made using different manufacturing parameters (confidential details), creating varying porosity levels in the initial sintered state—designated as Batch 1 and Batch 2 [61]. Batch 1 results were previously published in Reference 50. To minimise porosity in Batch 2, those samples underwent HIP treatment at 1160 °C under 104 MPa pressure for 4 h in an inert environment, creating a third group called Batch 2 + HIP. The two batches used different powder feedstocks, with Batch 2 powder containing more silica (SiO_2_) inclusions than Batch 1. Given this difference, the study primarily compares Batch 2 versus Batch 2 + HIP results, with occasional references to Batch 1 data. Table 2 presents the porosity fraction (f_p_) values for the different specimen batches studied. Batch 2 showed significantly lower porosity compared to Batch 1 due to optimised processing parameters that improved green body properties. HIP of Batch 2 samples further decreased porosity to approximately 1%, though it didn’t eliminate all pores. In the as-sintered state, porosity levels varied depending on the build orientation during manufacturing. HIP was employed to enhance mechanical performance, with particular emphasis on evaluating its effectiveness in improving fatigue resistance. The HIP treatment successfully decreased porosity levels.

**Table 2:** Porosity levels and mechanical tensile characteristics of various binder jetting process 316L specimen batches investigated in this research [62].

Batch	Orientation	f_p_ (%)	σ_y_ (MPa)	σ_u_ (MPa)	e_f_ (%)
1 [63]	ZX	4.655 ± 0.78	190 ± 17	548 ± 10	67.6 ± 7
	XY	4.2675 ± 0.43	193 ± 12	553 ± 4.4	76 ± 6.7
2	ZX	2.06 ± 0.12	226 ± 3.3	575 ± 9.5	64 ± 7
	XY	2.55 ± 0.31	224 ± 2.8	570 ± 6.1	76 ± 6.7
2 + HIP	ZX	1.15 ± 0.05	205 ± 5.6	556 ± 2.7	98 ± 6
	XY	1.11 ± 0.05	199 ± 3.34	551 ± 3	93.6 ± 5.6
CM [63]	-	0	273 ± 4	622 ± 6	65 ± 10

To consolidate the findings presented above, Table 3 provides a comparative summary of the mechanical properties of stainless steel 316L fabricated using various AM techniques under different post-processing conditions, based on representative data from the literature.

**Table 3:** Summary of mechanical properties of stainless steel 316L fabricated via different AM methods and post-processing Conditions.

AM Method	Post-Processing	UTS (MPa)	YS (MPa)	Porosity (%)	Reference
L-PBF	As-built	~560–650	~480–540	<1.5	[2,8]
L-PBF	Heat-treated	~700–800	~600–720	<1.5	[52,53]
L-PBF	HIP + Heat-treated	~750–820	~610–730	~0.1	[60,62]
Binder Jetting	Sintered (1380 °C)	~473.3	~182.7	~2–5	[15]
Binder Jetting	Sintered (1400 °C)	~387.0	~247.6	>5	[15]
DED	As-built	~500–600	~380–450	~1–2	[22,24]
DED	Functionally graded + heat treated	~650–730	~500–600	~1–2	[32]

### 3.3. Surface Finishing

The surface condition of stainless-steel components determines performance in forming operations. Surface roughness influences numerous tribological factors, including frictional stability, lubricant film retention, and wear resistance, all impacting dimensional accuracy, component life, and forming energy requirements. As-built surfaces produced via L-PBF and DED exhibit roughness values exceeding 10–25 µm (R_a_). This elevated roughness results from balling, partially melted particles, melt pool spatter, and layer staircase effects. Such surface features function as stress concentrators, reduce contact uniformity, and may lead to premature cracking or delamination in formed components [64,65].

**Figure 18:**
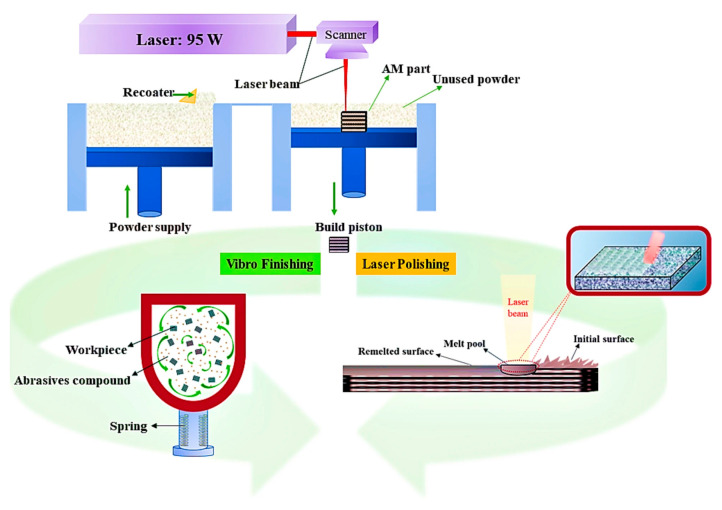
Schematic of the vibro-fnishing and laser-polishing post-surface treatment processes [70].

**Figure 19:**
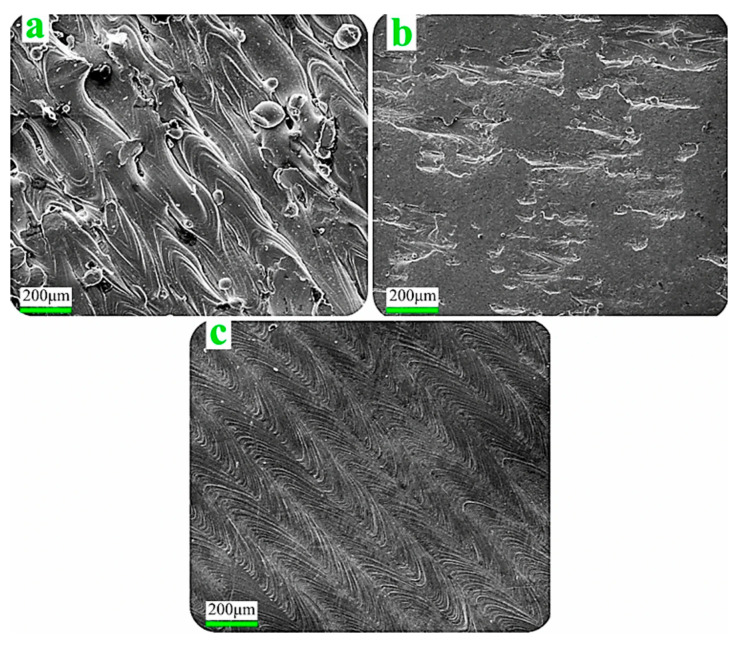
Tailoring surface characteristics of laser powder bed fusioned AISI 316L stainless steel for biomedical applications: (**a**) SLM-as, (**b**) SLM-VF, and (**c**) SLM-LP samples [70].

Several post-processing surface finishing techniques address these limitations by smoothing surfaces, removing adhered powder residues, and minimising micro-notches. LSR utilises re-scanning laser technology to locally melt and re-solidify the top surface layer, flattening irregularities and homogenising surface topography. LSR additionally reduces residual stress and surface porosity [66]. Electropolishing involves electrochemical removal of surface asperities using acid electrolytes, producing smooth, corrosion-resistant surfaces. This technique proves effective for 316L stainless-steel, reducing R_a_ values below 1 µm [67]. AFM employs semi-viscous abrasive-laden media extruded through internal or external surfaces to perform controlled micro-scale polishing. This method effectively smooths inaccessible geometries in dies or internal tool cavities [68]. Post-processing methods including vibro-finishing (VF) and laser-polishing (LP) were applied to improve the surface quality of the as-fabricated specimens, as illustrated in Figure 18. VF is a mechanical post-processing method that places parts in a vibrating chamber filled with abrasive media. The combined action of vibration and media effectively removes surface irregularities, burrs, and partially fused particles, resulting in smoother surfaces and reduced roughness. VF is especially suitable for complex geometries produced by additive manufacturing, providing uniform surface improvement without aggressive material removal. Comparative studies have shown that VF performs favorably alongside other techniques such as finish machining and drag finishing, particularly in enhancing surface quality and fatigue performance of AM-fabricated stainless steels [69]. Post-processing ensured pore connectivity for biological integration, confirming L-PBF’s suitability for functional, load-bearing bone scaffolds. Patient-specific dental crowns and implants are designed from intraoral scanning data and exhibit complex anatomical geometries tailored to individual patients. Figure 19a–c present the surface topography of the specimens’ upper surfaces. The as-built SLM samples display adhered loose powder particles and solidified ripple formations on their surfaces. These characteristics result in compromised interlayer adhesion, elevated surface roughness, and reduced material density. The presence of partially fused particles indicates that dispersed powder within the build chamber underwent melting and subsequently deposited onto the component surfaces. Furthermore, the periphery of each laser scan track solidifies initially, while the central region maintains a molten state and flows toward the active scanning zone due to surface tension effects. Examination of the surface morphology following VF and LP treatments reveals that both processes effectively eliminate spherical particles from the surface while creating a flattened topology. The surface irregularities primarily attributed to molten ripples are substantially reduced through both VF and LP methodologies, with these treatments significantly minimising surface valleys. Figure 19b demonstrates a marked transformation in the VF-treated surface morphology, exhibiting considerably reduced roughness compared to the as-built SLM specimen. The treated samples consistently show lower roughness values than the untreated counterparts. Evidence of plastic deformation and surface smoothing is clearly apparent, leading to distinct morphological changes. SEM analysis confirms that the LP-treated surface achieves superior smoothness relative to other specimens, corroborating the quantitative roughness measurements. Each technique offers advantages depending on component geometry, material type, and desired surface integrity.

Combined finishing approaches achieve micro-scale precision, especially for forming dies used in ultrathin sheet metal forming, where frictional consistency and surface quality remain paramount. Despite advances in AM resolution, finish machining remains necessary to meet tight dimensional tolerances, edge sharpness, and surface curvature precision required for forming tools such as micro dies, forming punches, and progressive stamping inserts. CNC milling, grinding, electro-discharge machining (EDM), and honing are frequently employed post-AM to refine component geometry. Hybrid manufacturing systems perform additive fabrication and subtractive finishing within the same platform, reducing post-processing steps while ensuring alignment accuracy. This approach proves particularly advantageous when producing tools with conformal cooling channels, graded structures, or multi-material interfaces [70,71]. Hybrid processing improves manufacturability while shortening lead times and ensuring tool readiness. As AM technologies evolve, integrated post-processing combining surface conditioning, thermal densification, and high-precision machining will prove critical for ensuring AM stainless-steel components meet demanding requirements of forming-intensive applications.

## 4. Challenges and Innovations

Despite the substantial advancements in AM of stainless-steel, several critical challenges remain that hinder its widespread adoption in high-precision and forming-intensive applications. These challenges span from mechanical limitations in part performance to process variability, material sustainability, and economic scalability. Ongoing research has introduced innovative solutions to address these limitations, including novel alloy development, intelligent control systems, and environmentally conscious process redesigns.

### 4.1. Formability Challenges in AM Stainless-Steels

Although AM enables fabricating geometrically complex and functionally optimised stainless-steel components, its application in forming-intensive processes remains limited by intrinsic formability challenges. Poor formability in AM-fabricated stainless-steels stems from residual stress accumulation, limited elongation and ductility, and mechanical anisotropy.

Laser-based AM processes such as L-PBF and DED employ localised, layer-wise energy input that induces steep thermal gradients, resulting in rapid cooling and solidification. These thermal cycles create large temperature differentials between newly deposited layers and underlying substrates, leading to tensile residual stress accumulation throughout components. These residual stresses prove detrimental to subsequent forming applications, serving as stress concentrators that promote crack initiation and propagation during drawing and bending operations [72]. The accumulated stresses lead to geometric distortion and interlayer delamination following support structure removal, compromising dimensional stability [73]. Additionally, these stresses result in premature tooling failure and unpredictable springback behaviour, affecting manufacturing reliability and dimensional accuracy [74]. Standard annealing may not fully eliminate residual stresses, particularly in thick-walled or geometrically complex components, necessitating advanced stress mitigation techniques such as multi-step annealing, HIP, and laser-based remelting. While AM stainless-steels such as 316L or 17-4PH typically exhibit high yield strength due to fine cellular microstructures, they suffer from significantly low ductility and elongation to fracture.

Empirical studies report elongation values for 316L components ranging from 25–35%, compared with approximately 50% for annealed wrought material. This deficiency can result in wrinkling, tearing, and fracture at low strain paths during deep drawing, and reduced fatigue resistance under cyclic forming loads. Mechanical anisotropy arises from layer-by-layer deposition strategies that promote directional grain growth and texture development along Z-axis. This creates substantial mechanical performance differences across orthogonal directions. Lower tensile strength and ductility typically occur in Z-direction compared with XY-plane orientations [75]. Directional dependence in strain hardening leads to inconsistent material flow and non-uniform thickness distribution during forming [76]. This anisotropy complicates AM component use in multi-directional deformation environments, such as stamping dies, punches, or medical microcomponents where dimensional uniformity and repeatable performance remain crucial. Without targeted heat treatment or recrystallisation, these orientation-dependent properties pose major reliability concerns.

### 4.2. Innovative Solutions and Emerging Techniques

To address formability and performance limitations inherent in additively manufactured stainless-steels, a comprehensive suite of scientific and engineering solutions has emerged. These innovations target unique challenges including residual stress, ductility loss, anisotropy, and variability in as-built components. This section outlines advancements in AM-specific alloy design, real-time process monitoring, AI-driven parameter optimisation, and advanced post-processing methods that enable AM evolution into production-grade solutions.

Conventional stainless-steel compositions such as 316L and 17-4PH were not originally developed for thermal gradients, cooling rates, and solidification dynamics characteristic of AM processes. Consequently, AM-specific alloy design has become a growing research priority, focusing on microsegregation suppression via controlled Cr/Ni/Mo ratios, grain refinement using additions like Nb, Ti, or Zr, and improved solidification behavior using trace elements [77]. Nb-modified 316L alloys demonstrate enhanced isotropy and crack resistance during L-PBF, while compositional tuning in 17-4PH has led to improved aging response and reduced interlayer porosity [78]. These material-level innovations prove vital for improving AM component formability and fatigue resistance. In-situ monitoring technologies have become critical tools for maintaining process stability and ensuring component quality during fabrication. These systems include thermal imaging for melt pool diagnostics, photodiode arrays and optical tomography for surface defect mapping, and acoustic emission for detecting delamination [79]. These sensors provide data to closed-loop control systems that autonomously adjust energy input, scan patterns, or recoating parameters in real time. Melt pool size fluctuations can trigger immediate laser power recalibration to maintain deposition consistency—an approach shown to reduce porosity and warping in stainless-steel builds by over 50% [80]. AI and machine learning models are now extensively applied to streamline AM process development and reduce costs. These applications include supervised learning algorithms trained on image data to classify defects [81], predictive models correlating process parameters with outcomes such as residual stress, anisotropy, or mechanical strength [82], and reinforcement learning frameworks for autonomous process parameter tuning across multiple build geometries and materials. Recent studies using convolutional neural networks demonstrate over 90% accuracy in identifying pore formation and predicting surface roughness trends during L-PBF of 316L [83]. These tools enable intelligent compensation strategies and form the foundation for digital twins, which simulate thermal-mechanical evolution of components during AM processing.

Post-processing strategies continuously evolve to address as-built stainless-steel AM component limitations. Key developments include functionally graded heat treatments, where spatially variable annealing enhances ductility in flexible zones while preserving strength in load-bearing regions. Custom HIP cycles, including controlled cooling ramps and high-pressure dwell stages, are optimised to eliminate specific defect morphologies [84]. Targeted LSR improves microstructure at fatigue-prone edges without bulk property alteration. Thermo-mechanical cycling combines low-strain deformation and subcritical annealing to achieve equiaxed grains and suppress textural anisotropy [85]. These techniques have increased elongation by up to 40% and reduced tensile anisotropy in 17-4PH stainless-steel tools, particularly those subjected to high-cycle forming operations [86].

## 5. Conclusions

AM has emerged as a transformative pathway for producing stainless-steel components with intricate geometries, tailored microstructures, and multifunctional capabilities. Through advanced techniques such as L-PBF, binder jetting, and DED, AM enables unprecedented control over component design, material utilisation, and functional integration.

This review highlights recent progress in stainless-steel microfabrication strategies, emphasising their suitability for forming tools, biomedical scaffolds, high-temperature energy components, and precision automotive systems. Microstructural control through process parameter optimisation, post-processing protocols including heat treatment, HIP, and surface finishing methods prove critical for overcoming limitations associated with mechanical anisotropy, residual stress accumulation, and reduced ductility. Despite these advances, several challenges persist. The inherent anisotropy, limited elongation, and surface roughness of components continue impeding widespread deployment in high-load forming operations. Innovative solutions converge to address these limitations, including AM-specific alloy design, functionally graded structures, in-situ monitoring systems, and AI-driven adaptive control. These innovations enhance component performance while supporting scalable, intelligent manufacturing systems capable of real-time feedback and process correction.

The successful industrialisation of AM stainless-steel components in forming technologies depends on continued progress across three key domains: materials innovation, intelligent process control, and sustainable manufacturing practices. Through coordinated advances across these areas, AM is a core manufacturing strategy for high-performance stainless-steel components across biomedical such as biomedical micromachines, aerospace, energy, and manufacturing sectors.
